# Experimental carotid baroreceptor stimulation reduces blood flow velocities in the anterior and middle cerebral arteries of healthy individuals

**DOI:** 10.1186/s12576-023-00871-7

**Published:** 2023-06-13

**Authors:** Gustavo A. Reyes del Paso, Casandra I. Montoro, J. Richard Jennings, Stefan Duschek

**Affiliations:** 1 Department of Psychology, University of Jaén, 23071 Jaén, Spain; 2 Department of Psychiatry, School of Medicine, University of Pittsburgh, Pittsburgh, USA; 3 UMIT TIROL - University for Health Sciences and Technology, Hall in Tirol, Austria

**Keywords:** Baroreceptor, Blood pressure, Heart rate, Cerebral blood flow, Transcranial Doppler sonography

## Abstract

This study investigated effects of experimental baroreceptor stimulation on bilateral blood flow velocities in the anterior and middle cerebral arteries (ACA and MCA) using functional transcranial Doppler sonography. Carotid baroreceptors were stimulated by neck suction in 33 healthy participants. Therefore, negative pressure (− 50 mmHg) was applied; neck pressure (+ 10 mmHg) was used as a control condition. Heart rate (HR) and blood pressure (BP) were also continuously recorded. Neck suction led to reductions in bilateral ACA and MCA blood flow velocities, which accompanied the expected HR and BP decreases; HR and BP decreases correlated positively with the ACA flow velocity decline. The observations suggest reduction of blood flow in the perfusion territories of the ACA and MCA during baroreceptor stimulation. Baroreceptor-related HR and BP decreases may contribute to the cerebral blood flow decline. The findings underline the interaction between peripheral and cerebral hemodynamic regulation in autoregulatory control of cerebral perfusion.

## Background

The baroreceptors are stretch-sensitive receptors mainly located in the carotid sinus, aortic arch and lungs, which contribute to blood pressure regulation by reflex modulation of heart and vascular resistance. Within a negative feedback loop (i.e., the baroreflex), blood pressure fluctuations cause changes in the firing rate of the baroreceptors, which via connections with brainstem structures precipitate compensatory adjustments of heart rate, ventricular contractility and vasomotor activity [8].

In addition to effects on cardiovascular function, activation of the baroreceptors may also modulate central nervous system activity. Experimental stimulation of the carotid baroreceptors by neck suction (which increases transmural pressure and stretches the baroreceptors, simulating a blood pressure rise) has led to reduction of cortical excitability [36], attenuation of pain perception [9, 39] and decrease of somatic muscle tone and sensorimotor performance [19, 32]. Although the state of research is not fully consistent (see [20, 21], it is commonly acknowledged that baroreceptor activation causes a generalized inhibitory effect on higher brain areas [15, 16, 37].

The present study examines possible effects of experimental baroreceptor stimulation on cerebral blood flow. Such effects may occur due to direct interactions between peripheral and cerebral hemodynamics. Heart rate deceleration and reductions of contractility and vascular tone following baroreceptor activation trigger blood pressure decline, which in turn may decrease brain perfusion. This hypothesis is disputable insofar as it is commonly assumed that autoregulatory mechanisms keep cerebral blood flow virtually constant by buffering systemic blood pressure fluctuations [22]. To stabilize perfusion, cerebral microvessels constrict during increases, and dilate during decreases, in blood pressure. However, recent studies have challenged the complete independence of cerebral blood flow from blood pressure (e.g., [30]. Spontaneous blood pressure fluctuations, as well as those elicited by physical manipulations, to certain degree are transferred to brain perfusion (e.g., [3, 31, 33]. Moreover, blood pressure rise during exercise and cognitive activity is accompanied by increased cerebral blood flow [13, 14, 35]. However, the degree of blood pressure decline elicited by baroreceptor stimulation required to reduce brain perfusion is still unknown [41].

In this study, functional transcranial Doppler sonography (fTCD) was applied to quantify changes in cerebral blood flow. This ultrasound technique enables continuous non-invasive recording of blood flow velocities in the basal cerebral arteries at a high temporal resolution [1, 10]. Bilateral recordings were made of flow velocities in the anterior and middle cerebral arteries (ACA and MCA), which supply a large part or the cerebral cortex. While the perfusion territory of the ACA consists of the medial cortical areas, the MCA supply most of the lateral parts of both hemispheres [24]. To evaluate possible impact of peripheral hemodynamics on cerebral blood flow, heart rate and blood pressure were also continuously recorded.

In a previous fTCD study, flow velocity in the right MCA was recorded during neck suction in healthy individuals [41]. Flow velocity did not change during acute baroreceptor stimulation but slightly decreased during baroreceptor resetting in a subgroup of participants. In another study, bilateral MCA flow velocity modulations were seen during oscillatory neck pressure [34]. The present study builds on this research, where bilateral recordings in the ACA and MCA, together with peripheral cardiovascular recordings during neck suction, are novel aspects.

The following two main hypotheses were tested: (1) Blood flow velocities in the ACA and MCA of both hemispheres will decrease during neck suction. (2) Blood pressure decline during baroreceptor stimulation will contribute to the reduction in cerebral blood flow. Positive associations between the magnitude of the blood pressure response to neck suction and the decreases in bilateral ACA and MCA flow velocities will support this hypothesis.

## Methods

### Participants

Taking a conservative effect size of 0.25, an alpha level of 0.05 and a beta error of 20% as a basis, a sample size of 24 participants is optimal to achieve statistical power above 80% in a repeated measures analysis of variance (ANOVA). As fTCD recordings of the ACA and MCA are not feasible in all individuals, 33 psychology students (26 women, 7 men) aged between 18 and 27 years were included in the study. None of them suffered from any cardiovascular disease (including hypertension) or was receiving pharmacological treatment affecting the cardiovascular or central nervous system. Each student received a course credit for her/his participation.

### Baroreceptor manipulation

Carotid baroreceptors were mechanically stimulated with a laboratory-built pneumatic device under computer control. To account for possible effects of somatosensory perception on cerebral blood flow velocities [2, 29], slight neck pressure, which is not perceptually differentiable from neck suction, was applied as a control condition. A pump allowed for the production of negative and positive pressures, which by solenoid valves and tubes were transmitted to two Teflon chambers (5 cm height, 4 cm width in its internal dimension) positioned at the neck, above both carotid sinuses [28]. The chambers were fixed to the neck by a cuff. The carotid sinus bifurcations were identified by manual palpation of the carotid horns (boney protrusions within the carotid triangle of the neck). The suction condition involved negative pressure of − 50 mmHg,the control condition involved positive pressure of + 10 mmHg. Participants were administered eight trials of each baroreceptor condition alternating between suction and pressure; the starting condition was counterbalanced across participants. The duration of both conditions was 10 s (inter-trial intervals 45 s).

### Recording and analysis of cerebral blood flow

Cerebral blood flow velocities were measured by fTCD employing a digital Multi-Dop L2 (DWL Elektronische Systeme, Sipplingen, Germany). After vessel identification, recordings were conducted in the ACA and MCA of both hemispheres through the temporal bone windows, using two 2-MHz transducers probes fixed over the skull by a head harness. The ACA were insonated at a depth of 60–70 mm and the MCA at a depth of 48–55 mm. The spectral envelope curves of the Doppler signal were recorded at a sample rate of 100 Hz and resampled at 1 Hz. The analysis was based on the mean flow velocity index, which is less vulnerable to artifacts than systolic or diastolic flow velocity and demonstrates the highest correlation with blood volume streaming through a vessel [10].

The data were averaged across the eight trials of each condition, i.e. within neck suction and within neck pressure. Responses to baroreceptor manipulation were expressed as relative (percent) changes in flow velocity (dFV) with respect to baseline (FVbas) according to the formula dFV = (FV[t]—FVbas) *100/FVbas, where FV(t) is the flow velocity over the course of time. The mean flow velocity during the 10 s before neck suction/pressure served as baseline and the following 13 s as observation period.

### Recording and analysis of cardiovascular parameters

A Task Force Monitor (CNSystems, Graz, Austria) was used for continuous beat-to-beat heart rate and blood pressure recordings. Four electrodes were applied to the chest, two at the shoulders and two at the lower rib cage (Einthoven I and II), to record two bipolar electrocardiograms (ECGs). Continuous measurements of mean arterial pressure (MAP, indexed as average pressure during the cardiac cycle) were taken from the first phalange of the second and third fingers of the right hand. The hand was positioned at the level of the heart. In addition, oscillometric blood pressure was taken from the left brachial artery. The Task Force Monitor recalibrates continuous finger blood pressure according to brachial artery blood pressure every 60 s without interrupting recording. Sampling rate was 1000 Hz for ECG and 200 Hz for continuous finger blood pressure. Beat-by-beat data of heart rate and MAP were transformed into second-by-second values and expressed as difference scores with respect to the mean value of the baseline, i.e., the 10 s before the beginning of neck suction/pressure.

### Procedure

Participants were tested in a semi-prone position. Blood flow velocities in the two vessels were recorded sequentially because it is not possible to reliably measure flow velocities in both ACA and MCA simultaneously. This limitation led us to repeat the entire procedure twice, once for each pair of arteries. The artery starting order (ACA vs. MCA) was counterbalanced across participants. Insonation of the ACA and/or MCA was not feasible in some participants; therefore, the following recordings were available for analysis: left ACA in 31 participants, right ACA in 30 participants, left MCA in 30 participants, right MCA in 31 participants. Participants were asked to avoid movements during recordings. They were previously requested to refrain from vigorous exercise, smoking and drinking alcohol or beverages containing caffeine for 2 h before the experimental session. The study protocol was approved by the Bioethics Committee of the University of Jaén; all participants provided their written informed consent.

### Statistical analysis

Data were analyzed by repeated measures ANOVA. For blood flow velocity a 2 × 2x13 design was applied for each artery (ACA and MCA) with the following repeated measures factors: Condition (neck suction vs. neck pressure), Hemisphere (left vs. right) and Time (the 13 values of blood flow velocity, i.e., 13 s observation period). As two sets of cardiovascular parameters (heart rate, MAP) were available from the recording as it was duplicated for each pair of arteries, second-by-second values were first aggregated over the two recordings for the ACA and MCA. For cardiovascular variables a 2 × 11 ANOVA was used, with Condition and Time as repeated measures factors. Here, the Time factor included the 10 s-by-second post-stimulus values plus a first zero value. The cardiovascular response to neck suction is so fast, especially for heart rate, that most of it is already manifested at the first second. Therefore, to fully capture its effect it deemed necessary to include a first baseline point (zero) to enable analysis of the change from baseline to second 1. The Huynh–Feldt epsilon correction was applied for the adjustment of degrees of freedom. Post-hoc tests were performed where appropriate. Paired samples t-tests (one-tailed) were computed to test for differences between the neck suction and neck pressure conditions for each second of the response. Moreover, paired samples t-tests (one-tailed) were also performed to compare the values of the variables for each second of the response with respect to the baseline. Relationships between cardiovascular parameters and blood flow velocities were quantified by means of Pearson correlations. In the computation of the correlations, second by second values of heart rate and MAP were related to ACA and MCA flow velocities of the same second (time lag 0), and the following (time lag 1) and next but one (time lag 2) seconds.

## Results

Table 1 includes the baseline values of ACA and MCA blood flow velocities and those of heart rate and MAP obtained during the ACA and MCA recordings. Baseline heart rate and MAP did not differ between the two data sets [F(1, 30) = 0.087, p = 0.77, µ^2^ = 0.003 for heart rate; F(1, 30) = 0.67, p = 0.42, µ^2^ = 0.022 for MAP].

**Table 1:** Baseline ACA and MCA blood flow velocities: heart rate and MAP values obtained during ACA and MCA baseline recordings (mean ± SD)

	ACA	MCA
Left flow velocity (cm/s)	49.06 ± 12.56	63.85 ± 15.78
Right flow velocity (cm/s)	43.19 ± 10.42	64.09 ± 15.39
Heart rate (bpm)	73.62 ± 9.72	72.92 ± 10.41
MAP (mmHg)	79.52 ± 12.07	80.28 ± 13.50

*ACA* anterior cerebral artery; *MCA* middle cerebral artery; *MAP* mean arterial pressure

Moreover, heart rate and MAP did not differ between the ACA and MCA recordings during the neck suction [F(1,30) = 0.25, p = 0.62, µ^2^ = 0.008 for heart rate; F(1,30) = 2.41, p = 0.13, µ^2^ = 0.075 for MAP] and neck pressure [F(1,30) = 0.09, p = 0.76, µ^2^ = 0.003 for heart rate; F(1,30) = 0.60, p = 0.44, µ^2^ = 0.020 for MAP] conditions. This justified the aggregation of the values over the two data sets.

### Effects of baroreceptor manipulation on cerebral blood flow velocities

**Fig. 1:**
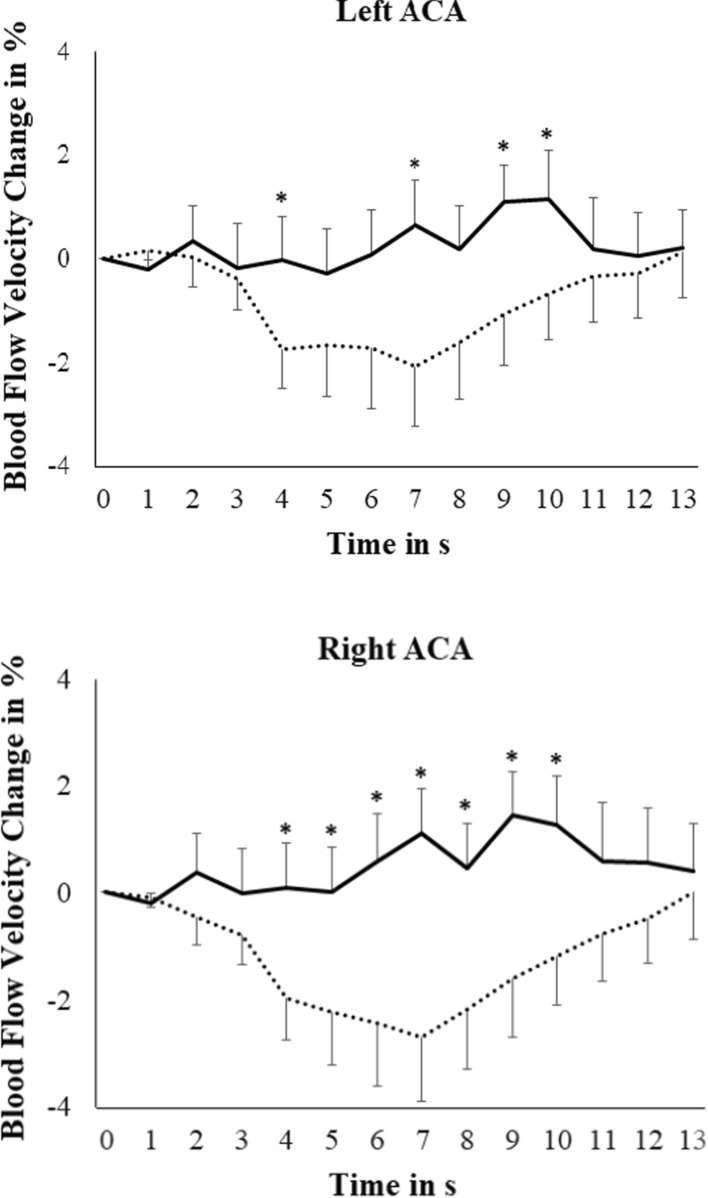
Grand averages of blood flow velocity changes in the left and right anterior cerebral arteries (ACA) during baroreceptor manipulation (doted line represents neck suction condition, solid line represents neck pressure condition; bars represent standard error of the mean; * p < .05 in the comparison between neck suction and neck pressure)

**Fig. 2:**
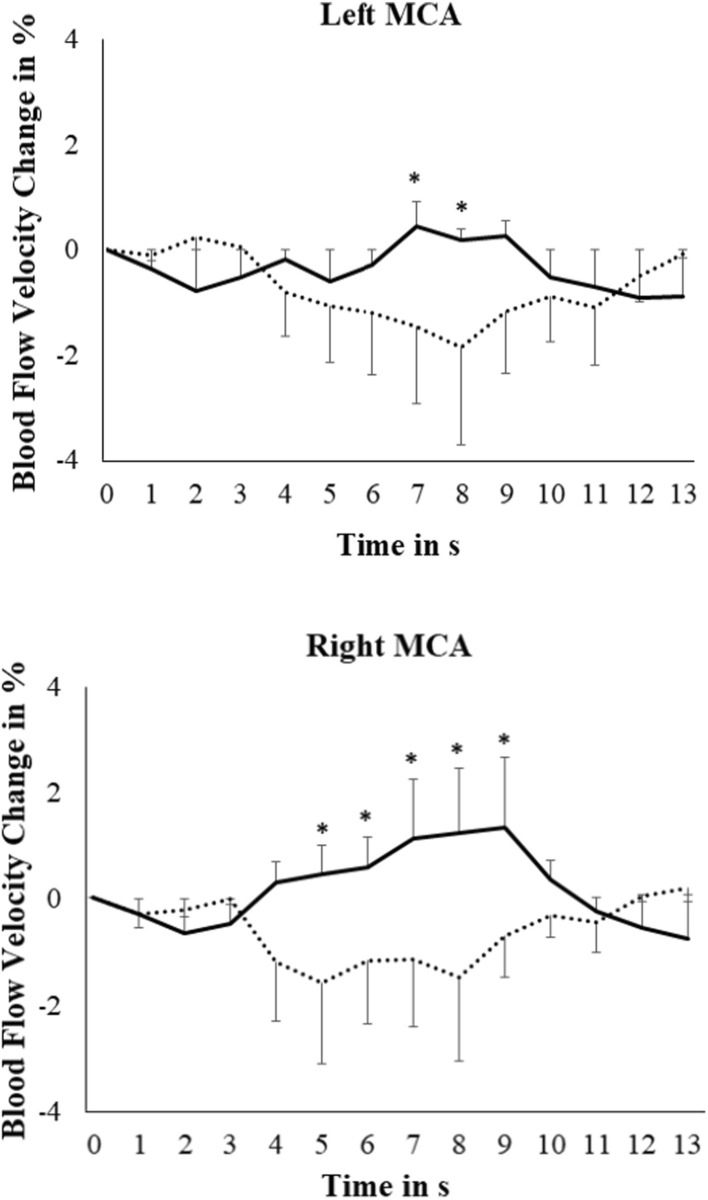
Grand averages of blood flow velocity changes in the left and right middle cerebral arteries (MCA) during baroreceptor manipulation (doted line represents neck suction condition, solid line represents neck pressure condition; bars represent standard error of the mean; * p < .05 in the comparison between neck suction and neck pressure)

Figures 1 and 2 display the changes in cerebral blood flow velocities in the ACA and MCA during the experimental procedures. The ANOVA pertaining to the ACA revealed a marginally significant Condition main effect [F(1, 29) = 3.41, p = 0.075, µ^2^ = 0.11]. However, the Time x Condition [F(5.49, 159.36) = 2.93, p = 0.012, µ^2^ = 0.092] and Condition x Hemisphere interactions [F(1, 29) = 5.96, p = 0.021, µ^2^ = 0.17] were significant. While neck suction produced a flow velocity decrease [F(3.30, 95.64) = 2.65, p = 0.048, µ^2^ = 0.084], no changes arose during neck pressure [F(5.35, 155.10) = 1.20, p = 0.31, µ^2^ = 0.040]. Overall, lower flow velocity was seen during neck suction than during neck pressure in the right hemisphere [F(1, 29) = 4.49, p = 0.043, µ^2^ = 0.13], but not in the left one [F(1, 29) = 2.31, p = 0.14, µ^2^ = 0.074].

According to post-hoc tests, the decrease from baseline during neck suction was significant from s 4 to s 8 in the right ACA [all ts(29) ≥ 1.97; all ps ≤ 0.029], and in s 4, s 5 and s 7 in the left ACA [all ts(30) ≥ 1.79; all ps ≤ 0.042]. Comparisons between the neck suction and neck pressure conditions revealed differences in s 4, s 7, s 9 and s 10 for the left ACA [all ts(30) ≤ -1.69; all ps ≤ 0.05], and from s 4 to s 10 for the right ACA [all ts(29) ≤ -2.13; all ps ≤ 0.024] (see Fig. 1).

In the MCA, no main effects arose, but the Time x Condition [F(3.91, 113.49) = 3.95, p = 0.005, µ^2^ = 0.12] and Time x Condition x Hemisphere interactions [F(4.09, 118.72) = 2.70, p = 0.033, µ^2^ = 0.085] were significant. The Time x Condition interaction was significant in both hemispheres, but the effect size was larger in the right [F(3.88, 116.48) = 4.10, p = 0.004, µ^2^ = 0.12] than in the left [F(3.47, 100.57) = 2.66, p = 0.044, µ^2^ = 0.084] hemisphere. During neck suction, flow velocities decreased in both hemispheres [F(4.60, 133.35) = 2.59, p = 0.033, µ^2^ = 0.082]. During neck pressure, significant effects of Hemisphere [F(1, 29) = 4.62, p = 0.040, µ^2^ = 0.14] and Time [F(5.81, 168.42) = 2.28, p = 0.040, µ^2^ = 0.073] and a Time x Hemisphere interaction [F(3.92, 113.56) = 2.87, p = 0.027, µ^2^ = 0.090] arose. In this condition, flow velocity increased in the right hemisphere [F(5.28, 158.48) = 3.23, p = 0.007, µ^2^ = 0.097] but not in the left hemisphere [F(4.87, 141.16) = 1.26, p = 0.29, µ^2^ = 0.042].

According to post-hoc tests, the decrease from baseline during neck suction was significant from s 4 to s 8 in the right MCA [all ts(30) ≥ 1.85; all ps ≤ 0.037], and from s 6 to s 8 and in s 11 in the left MCA [all ts(29) ≥ 1.87; all ps ≤ 0.036]. The increases during neck pressure in the right MCA were significant from s 7 to s 9 [all ts(30) ≥ -1.85; all ps ≤ 0.036]. Comparisons between the neck suction and neck pressure conditions revealed differences in s 7 and s 8 for the left MCA [all ts(29) ≤ -2.23; all ps ≤ 0.017], and from s 5 to s 9 for the right MCA (all ts(30) ≤ − 1.79; all ps ≤ 0.041) (see Fig. 2).

### Effects of baroreceptor manipulation on cardiovascular variables

**Fig. 3:**
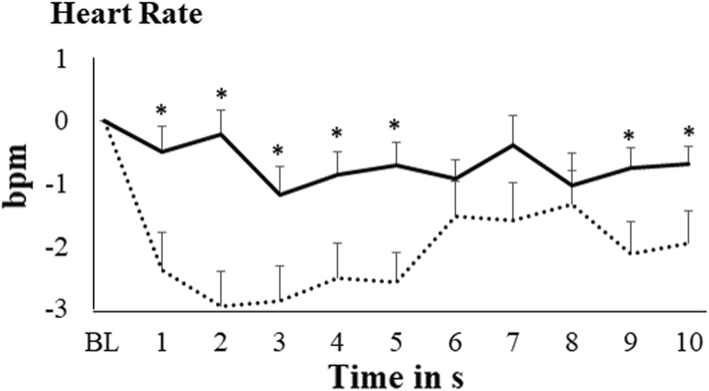
Changes in heart rate during baroreceptor manipulation (doted line represents neck suction condition, solid line represents neck pressure condition; bars represent standard error of the mean; *BL* baseline; * p < .05 in the comparison between neck suction and neck pressure)

**Fig. 4:**
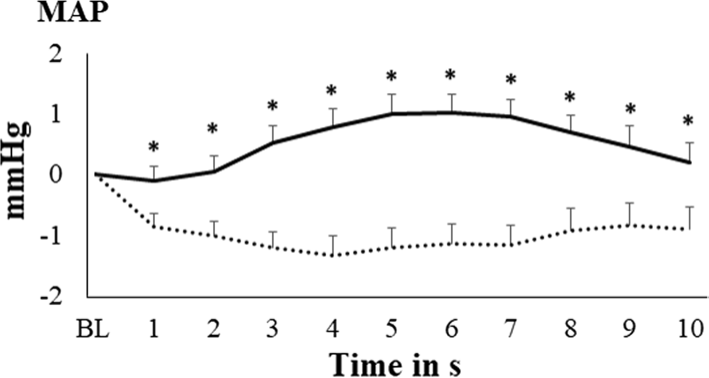
Changes in mean arterial pressure during baroreceptor manipulation (doted line represents neck suction condition, solid line represents neck pressure condition; bars represent standard error of the mean; *BL* baseline; * p < .05 in the comparison between neck suction and neck pressure)

Changes in heart rate and MAP are displayed in the Figs. 3 and 4. For the two variables, the Time x Condition interaction was significant [F(5.69, 182.06) = 3.25, p = 0.005, µ^2^ = 0.092 for heart rate; F(3.31, 106.03) = 8.49, p < 0.0001, µ^2^ = 0.21 for MAP]. While neck suction produced a heart rate decrease [F(5.63, 180.27) = 6.96, p < 0.0001, µ^2^ = 0.18], no heart rate change arose during neck pressure [F(5.70, 182.27) = 1.31, p = 0.26, µ^2^ = 0.039]. Neck suction was also associated with a decrease in MAP [F(2.65, 84.63) = 4.03, p = 0.013, µ^2^ = 0.112]; in contrast, MAP rose during neck pressure [F(3.43, 109.65) = 6.12, p < 0.0001, µ^2^ = 0.16].

According to post-hoc tests, heart rate and MAP decreases from baseline during neck suction were significant from s 1 to s 10 [all ts(32) ≤ − 2.34; all ps ≤ 0.012]. The MAP increase during neck pressure was significant from s 3 to s 8 [all ts(32) ≤ − 1.93; all ps ≤ 0.031]. Comparisons between neck suction and neck pressure revealed significant differences from s 1 to s 5, and from s 9 to s 10, for heart rate [all ts(32) ≤ − 2.17; all ps ≤ 0.018], and from s 1 to s 10 for MAP [all ts(32) ≤ − 2.46; all ps ≤ 0.001] (see Figs. 3 and 4).

### Correlations between cerebral blood flow velocities and cardiovascular variables

During the neck suction condition, heart rate in the seconds 2 to 8 correlated positively with left and right ACA flow velocities in the same seconds (time lag 0) and the following (time lag 1) and next but one (time lag 2) seconds (all rs ≥ 0.47; all ps ≤ 0.008). Moreover, heart rate in s 6 correlated positively with left MCA flow velocity in s 8 (time lag 2) (r = 0.36; p = 0.048); and heart rate in the seconds 6 and 7 correlated positively with right MCA flow velocities in the same seconds (time lag 0) and the following (time lag 1) and next but one (time lag 2) seconds (all rs ≥ 0.37; all ps ≤ 0.039).

Again during neck suction, MAP in s 3 correlated positively with left ACA flow velocity in s 5 (time lag 2) (r = 0.41; p = 0.021). Moreover, MAP in the seconds 4 to 8, correlated positively with left and right ACA flow velocities in the same seconds (time lag 0) and the following (time lag 1) and next but one (time lag 2) seconds (all rs ≥ 0.39; all ps ≤ 0.033); MAP in s 9 correlated positively with right ACA flow velocities in s 9 (time lag 0) and s 10 (time lag 1) (all rs ≥ 0.40; all ps ≤ 0.028); MAP in s 10 correlated positively with left and right ACA flow velocities in s 10 (time lag 0) and with left ACA flow velocity in s 11 (time lag 1) (all rs ≥ 0.38; all ps ≤ 0.039).

Correlations were also observed during the neck pressure condition. Heart rate in s 1 correlated positively with left MCA flow velocity in s 3 (time lag 2) (r = 0.44; p = 0.015) and left ACA flow velocity in s 2 (time lag 1) (r = 0.37; p = 0.042); moreover, heart rate in s 2 correlated positively with left and right MCA flow velocities in s 3 (time lag 1) and s 4 (time lag 2) (all rs ≥ 0.42; all ps ≤ 0.037); heart rate in s 7 correlated positively with left and right ACA flow velocities in the seconds 8 and 9 (time lags 1 and 2) (all rs ≥ 0.42; all ps ≤ 0.018); and heart rate in s 10 correlated positively with left ACA flow velocity in s 12 (time lag 2) (r = 0.47; p = 0.008).

**Fig. 5:**
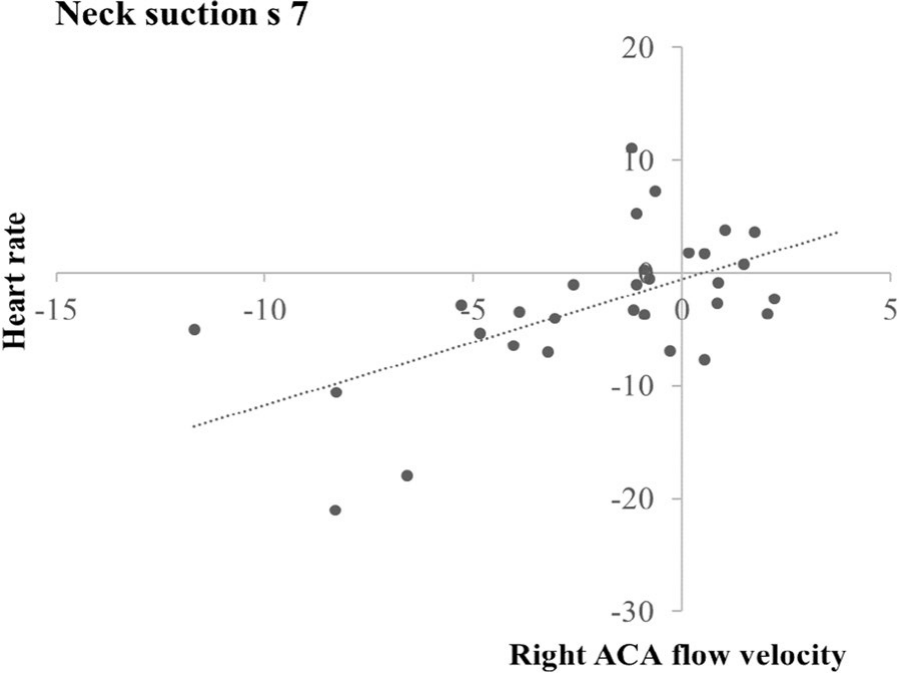
Scatter plot of the correlation between changes from baseline in heart rate and those in right ACA flow velocity during neck suction in s 7 (time lag 0)

**Fig. 6:**
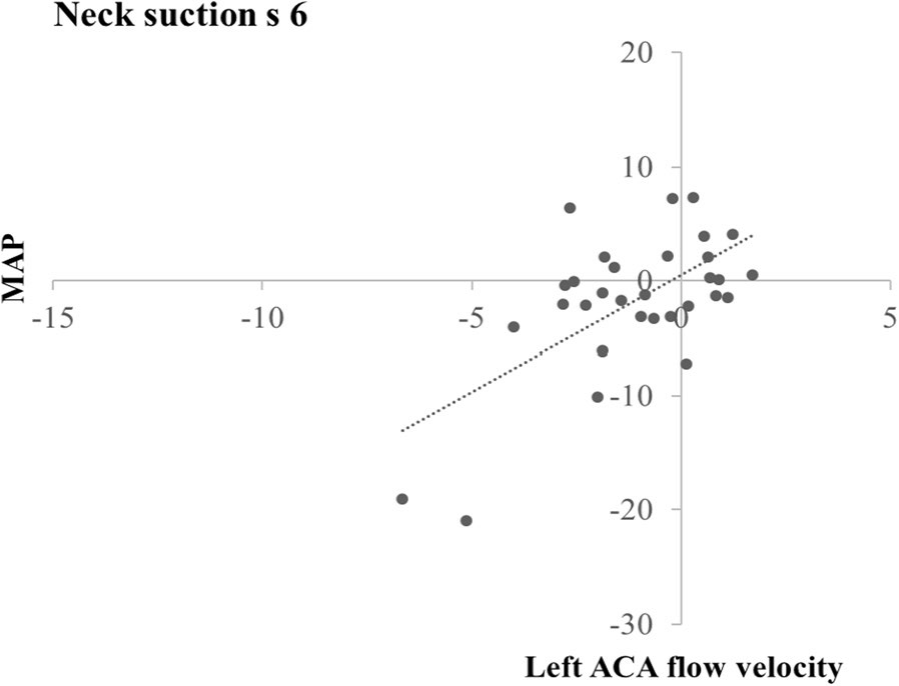
Scatter plot of the correlation between changes from baseline in MAP and those in left ACA flow velocity during neck suction in s 6 (time lag 0)

Figures 5 and 6 show the scatter plots for two exemplary correlations. Figure 5 illustrates the correlation between changes from baseline in heart rate and those in right ACA flow velocity during neck suction in s 7 (time lag 0, r = 0.59); and Fig. 6 illustrates the correlation between changes from baseline in MAP and those in left ACA flow velocity during neck suction in s 6 (time lag 0, r = 0.64).

## Discussion

This study investigated effects of experimental manipulation of the carotid baroreceptors in humans on cerebral blood flow velocities assessed by fTCD. While baroreceptor stimulation by neck suction led to decreases in flow velocities in the MCA and ACA of both hemispheres, neck pressure was associated with flow velocity increase in the right MCA. Heart rate and blood pressure decreased, as expected, during neck suction. During neck pressure, blood pressure increased, and heart rate remained unchanged. Moreover, the magnitudes of the declines in heart rate and blood pressure during neck suction correlated positively with the cerebral flow velocity decreases.

The observation of reductions of heart rate and blood pressure during neck suction reflects the basic mechanism of the baroreflex, i.e., compensatory cardiovascular adjustment to blood pressure fluctuations [8]. Baroreceptor afferents reach the nucleus of the solitary tract via the vagus and glossopharyngeal nerves. Projections from this structure innervate the nucleus ambiguus, the dorsal motor nucleus and the caudal and rostral ventrolateral medulla oblongata. The latter constitute the starting points of sympathetic and parasympathetic motoneurons, which execute the cardiovascular responses [7]. Neck suction acts on the underlying vessel wall to increase transmural pressure, signalling a blood pressure increase to the baroreceptors, neck pressure decreases transmural pressure signalling a blood pressure decrease. The small positive pressure induced in our study appeared sufficient to decrease transmural pressure and hence produced a reflex increase in blood pressure. This effect may be ascribed to the activation of the sympathetically-mediated vascular branch of the reflex, which triggers increases in peripheral resistance during blood pressure decreases [40]. The blood pressure rise during neck pressure occurred later than the heart rate response to neck suction, which reflects the known heightened latency of the sympathetic vascular response relative to the vagally transmitted cardiac response [40].

In accordance with our main hypothesis, cerebral blood flow velocities decreased during neck suction. Direct interactions between peripheral and cerebral hemodynamics may play a role in this observation, which is supported by the correlations between the changes in the assessed variables. The reflex heart rate and blood pressure decreases during neck suction correlated positively with the declines in bilateral ACA flow velocities; the decrease in heart rate also correlated with bilateral MCA blood flow velocity reductions. This finding contradicts the traditional assumption that in healthy individuals, cerebral autoregulation keeps cerebral perfusion independent of systemic hemodynamics, i.e., maintains cerebral blood flow independent of change in bodily blood pressure [22]. Various physiological processes mediate reduction of resistance of cerebral arterioles during blood pressure decline and increase of resistance during blood pressure rise. Due to myogenic mechanisms, changes in transmural pressure are directly followed by compensatory adjustment of vascular tone. Moreover, vascular tone changes according to alterations in CO_2_ partial pressure during increases and decreases in brain perfusion [26, 44]. Such changes would ensure the independence of cerebral perfusion from systemic blood pressure. However, there is evidence challenging the dissociation between peripheral and cerebral hemodynamics [30, 33]. For example, healthy subjects with chronically low blood pressure exhibited smaller increases in MCA blood flow velocities during cognitive activity than those with normal blood pressure [11, 17, 18]. Pharmacologically induced blood pressure enhancement led to increase of tonic MCA perfusion and stronger flow velocity responses during cognition [12]. Moreover, in healthy normotensive samples cognitively induced increases in heart rate and blood pressure correlated positively with simultaneously recorded MCA flow velocity increases [13, 14]. Spontaneous oscillations of peripheral blood pressure in various frequency ranges have been found closely associated with oscillations in ACA and MCA flow velocities [31]. These reports are in line with the correlations between the declines in blood pressure and ACA flow velocities from the present study. This supports the hypothesis that blood pressure reduction due to baroreceptor stimulation contributes to the decrease in cerebral blood flow velocities.

Correlations with blood pressure during neck suction were seen for flow velocities in the ACA but not in the MCA, which is difficult to explain. The ACA and MCA both arise from the internal carotid artery, such that similar effects of systemic blood pressure changes may be expected; however, the diameter and perfusion territory of the ACA are far smaller than those of the MCA. Therefore, a blood pressure decrease may have a greater impact on flow velocities in the ACA. Moreover, lower variability of the MCA response associated with its lower amplitude may have reduced the correlations. Changes in heart rate during the neck pressure condition correlated positively with ACA and MCA flow velocity modulations. However, heart rate changes during this condition were only small and unsystematic. Therefore, it is likely that the correlations between heart rate and flow velocity changes reflect the association of spontaneous heart rate fluctuation with those in ACA and MCA flow velocities [31], rather than the transmission of baroreceptor-related hemodynamic changes to cerebral perfusion.

In addition to direct interactions between peripheral and cerebral hemodynamics, cortical deactivation due to increased activity of baroreceptor afferents may be relevant to the decrease of cerebral blood flow velocities seen during neck suction [15, 16, 36, 37]. While the reflex adjustment of cardiovascular function to blood pressure fluctuations is confined to the brain stem, the involved medullary units are closely connected with higher brain regions [15, 38]. The nucleus of the solitary tract, which receives direct input from the baroreceptors, constitutes the starting point of afferents to the parabrachial nucleus in the pons, hypothalamus and periaqueductal gray. These structures in turn are closely linked to the amygdala, insula, cingulate cortex and medial prefrontal cortex [4, 5, 7]. Neural processes, brain metabolism and blood perfusion are closely connected (i.e., neurovascular coupling). This mechanism facilitates adjustment of local cerebral blood flow to neural activity through vasomotor changes in cerebral arterioles and capillaries [27]. Therefore, it may be that baroreceptor-related cortical inhibition contributes to the reduction of ACA and MCA blood flow velocities.

The adjustment of diameters of cerebral microvessels to neural activation processes is mediated by multiple neurochemical factors. For example, neurotransmitters including acetylcholine and catecholamines exhibit vasoactive properties, with their release causing dilation of arterioles in the active tissue [26]. Moreover, glia cells release vasodilators such as CO_2_, K + and adenosine during neural activity [25], agonists produced in neurons and glia cells activate vasoactive agents including calcium and nitric oxide in the endothelium [23]. In contrast to cerebral arterioles, the diameters of the basal cerebral arteries remain virtually constant during neural activity [10, 42]. Therefore, the reductions of flow velocities in the ACA and MCA can be attributed to increased resistance of down-stream microvessels.

While the insula is supplied by the MCA, the cingulate and medial prefrontal cortex are part of the perfusion territory of the ACA [24], the decline in flow velocities during baroreceptor stimulation may relate to diminished activity in these structures. The medial prefrontal cortex and cingulate occupy a greater volume than the insula; as such, the proportion of the deactivated area supplied by the ACA is greater than that supplied by the MCA. This may explain the greater flow velocity reduction in the ACA than in the MCA, as reflected in the larger effect size of the time by condition interaction seen for the ACA (see Figs. 1 and 2). While marked flow decline arose in the MCA and ACA of both hemispheres, the response showed only slight lateralization to the right. The state of research concerning hemispherical dominance of the cortical processing of cardiovascular information is still controversial, whereas both left and right lateralization has been reported (see [6, 43].

A limitation of the study pertains to the low spatial resolution of fTCD [10]. Therefore, neuroimaging studies are clearly warranted to provide insight into the precise spatial distribution of perfusion changes during baroreceptor stimulation. Furthermore, respiratory parameters and the phase of the cardiac cycle in which the onset of neck suction occurred were not controlled. Due to the lack of a direct measure of central nervous activity, the findings do not allow conclusions to be drawn regarding the degree to which the observed blood flow velocity modulations related to changes in blood pressure or neural activity. To disentangle the roles of neurovascular coupling and peripheral hemodynamic factors in cerebral blood flow modulations arising during baroreceptor stimulation, simultaneous application of fTCD and electroencephalography (EEG), for example, may be beneficial in future research.

Our findings complement a previous fTCD study assessing the effects of experimental baroreceptor stimulation on right hemispherical MCA blood flow velocities in healthy men and women in different age groups [41]. While no flow velocity changes were seen during acute baroreceptor stimulation in this study, a slight flow reduction arose during baroreceptor resetting in a subgroup of participants (young and middle-aged women). Considering this, our study demonstrated for the first time decreased flow velocities in the ACA and MCA of both hemispheres during baroreceptor stimulation in men and women. The differences in results between the studies may be ascribed to methodological factors; in addition to the unilateral MCA assessment, the intensity of neck suction was lower (-40 mmHg), and the stimulation interval (30 s) was longer, in the study of Saeed et al. [41] than in the present one.

## Conclusions

In conclusion, this study revealed initial evidence of reduction of blood flow in the perfusion territory of the ACA and MCA due to experimental stimulation of the carotid baroreceptors. To a certain degree, reflex blood pressure decline may transfer to cerebral perfusion, which underlines the interaction between peripheral and cerebral hemodynamics that occurs despite autoregulatory stabilization of cerebral blood flow. In addition, inhibition of cortical activity arising from baroreceptor afferents might play a role, which, due to neurovascular coupling, may be accompanied by a reduction of brain perfusion.

## Data Availability

The datasets used and/or analyzed during the current study are available from the corresponding author on reasonable request.
